# Tattoo Pigment in an Intramammary Lymph Node Mimicking Breast Malignancy

**DOI:** 10.7759/cureus.30336

**Published:** 2022-10-15

**Authors:** Jayda Jung, Gesine Peters, Shaun Donovan, Gudrun Peters

**Affiliations:** 1 Radiology, Flinders University, Adelaide, AUS; 2 University of Tasmania School of Medicine, Royal Hobart Hospital, Hobart, AUS; 3 Pathology, Sonic Healthcare, Diagnostic Services, Hobart, AUS; 4 Radiology, I-MED Radiology Network, Regional Imaging Tasmania, Hobart, AUS

**Keywords:** case report, breast malignancy, breast calcification, tattoo pigment, intramammary lymph node

## Abstract

There are many patterns of microcalcification in mammography. Distinguishing between these patterns can be challenging. A malignant cause needs to be assessed through further diagnostic workup. We present a case of a 36-year-old BRCA1 mutation carrier, presenting with a small mass containing calcification on her screening mammogram. A vacuum-assisted biopsy under tomosynthesis guidance was performed and demonstrated an intramammary lymph node showing prominent extracellular black pigment. To our knowledge, this is the first case report of tattoo pigment mimicking breast malignancy on mammography.

## Introduction

Mammographic microcalcification has a wide range of etiologies, both malignant and benign [1]. Microcalcification with an intermediate or high probability of malignancy requires further diagnostic workup, such as in the case presented. Uptake of tattoo ink in an intramammary lymph node mimicking a mass lesion containing calcification has not been previously described, however, as the prevalence of females with tattoos increases, tattoo pigment needs to be considered as a potential differential diagnosis of breast calcification [2].

## Case presentation

**Figure 1:**
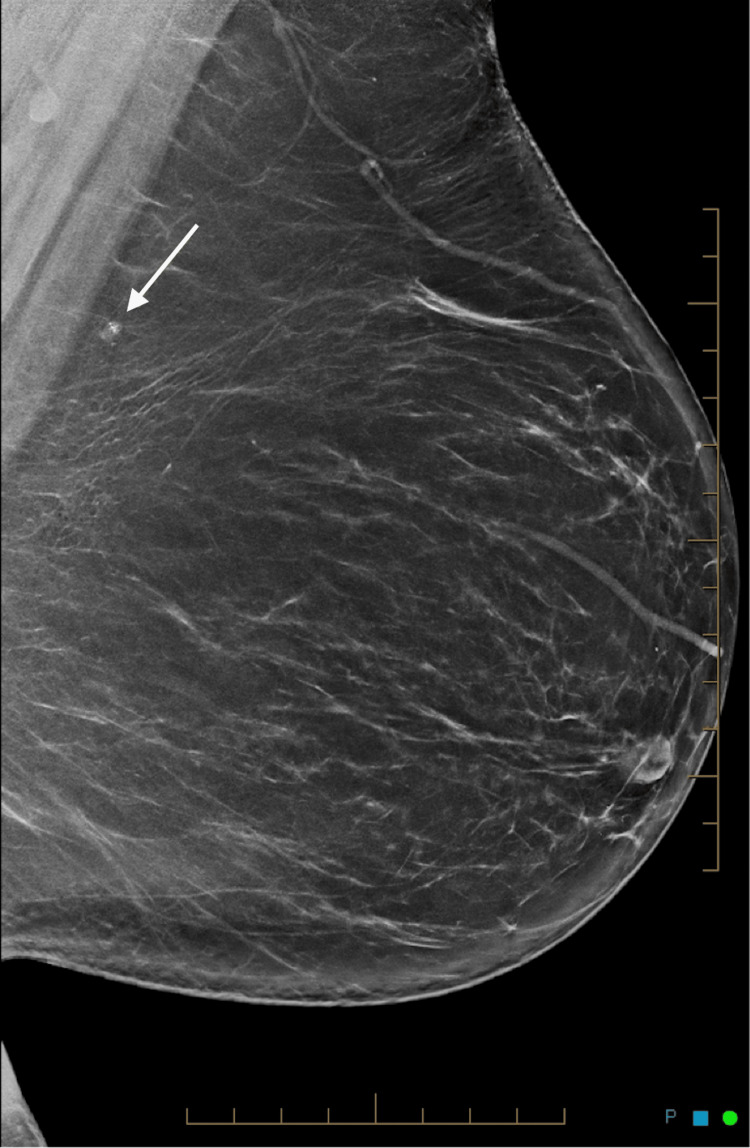
Left mediolateral oblique mammogram showing a small mass lesion containing radiopaque material.

**Figure 2:**
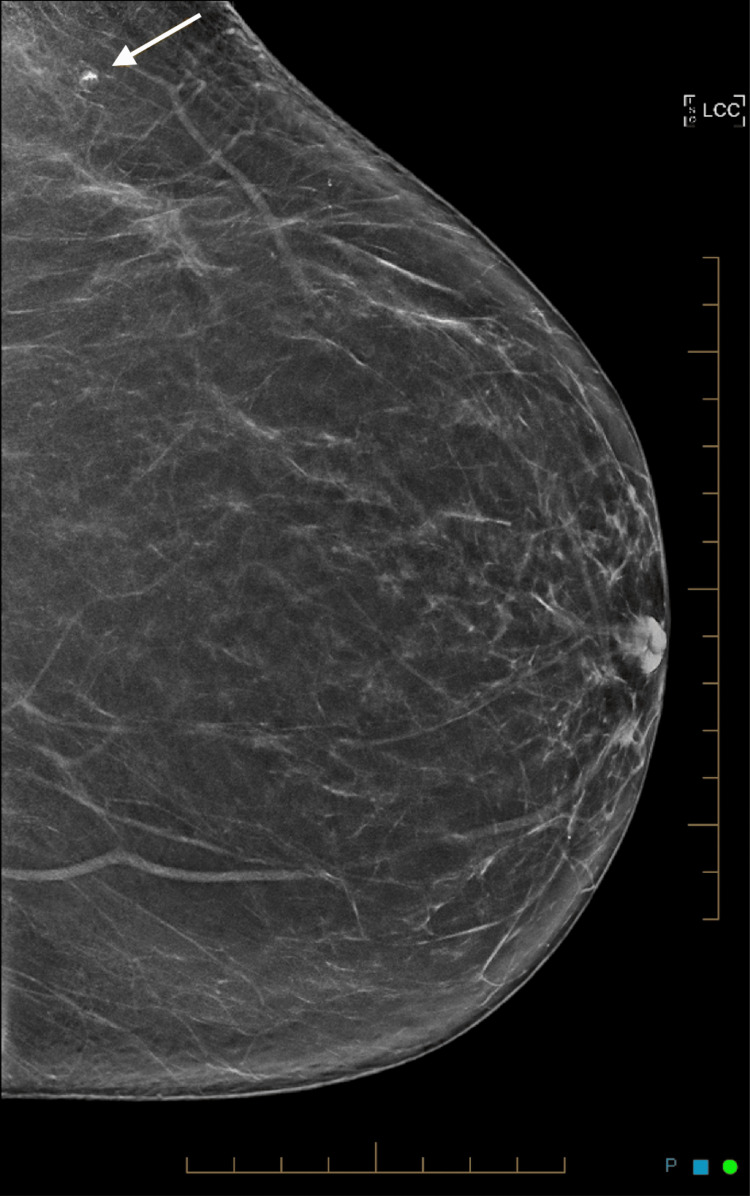
Left craniocaudal mammogram showing a small mass lesion containing radiopaque material.

**Figure 3:**
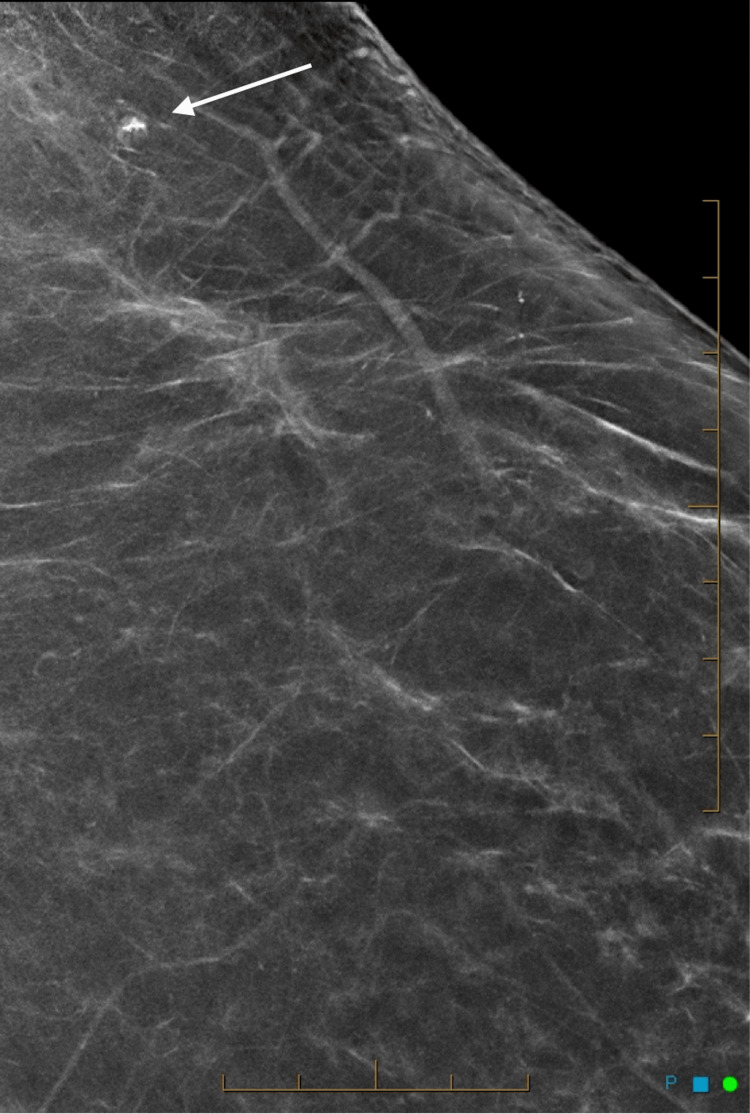
Spot magnification mammogram showing the small mass lesion containing radiopaque material.

A 36-year-old female, who is a known BRCA1 mutation carrier through genetic testing, presented for initial breast imaging surveillance. Mammography demonstrated fatty replacement of the breast tissue and a 5 mm mass lesion containing microcalcification at the 2 o’clock position, 140 mm from the left nipple (Figures 1-3).

The targeted left breast ultrasound did not demonstrate any abnormality. Therefore, a mammographic breast biopsy was performed using Hologic Selena Dimensions with the Affirm Breast Biopsy Guidance System and ATEC Vacuum-Assisted Breast Biopsy system with a 9-gauge needle.

**Figure 4:**
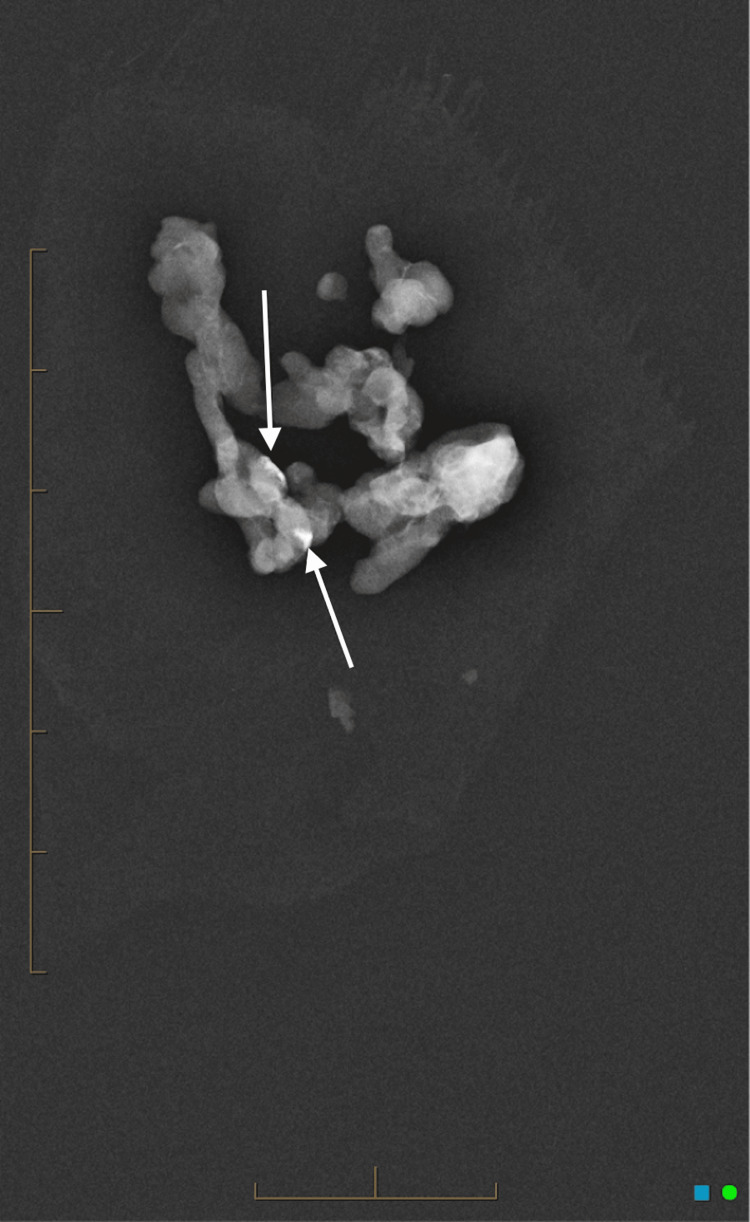
Specimen X-ray confirming radiopaque material within the specimen.

Post-biopsy specimen X-ray confirmed radiopaque material in the sample (Figure 4).

**Figure 5:**
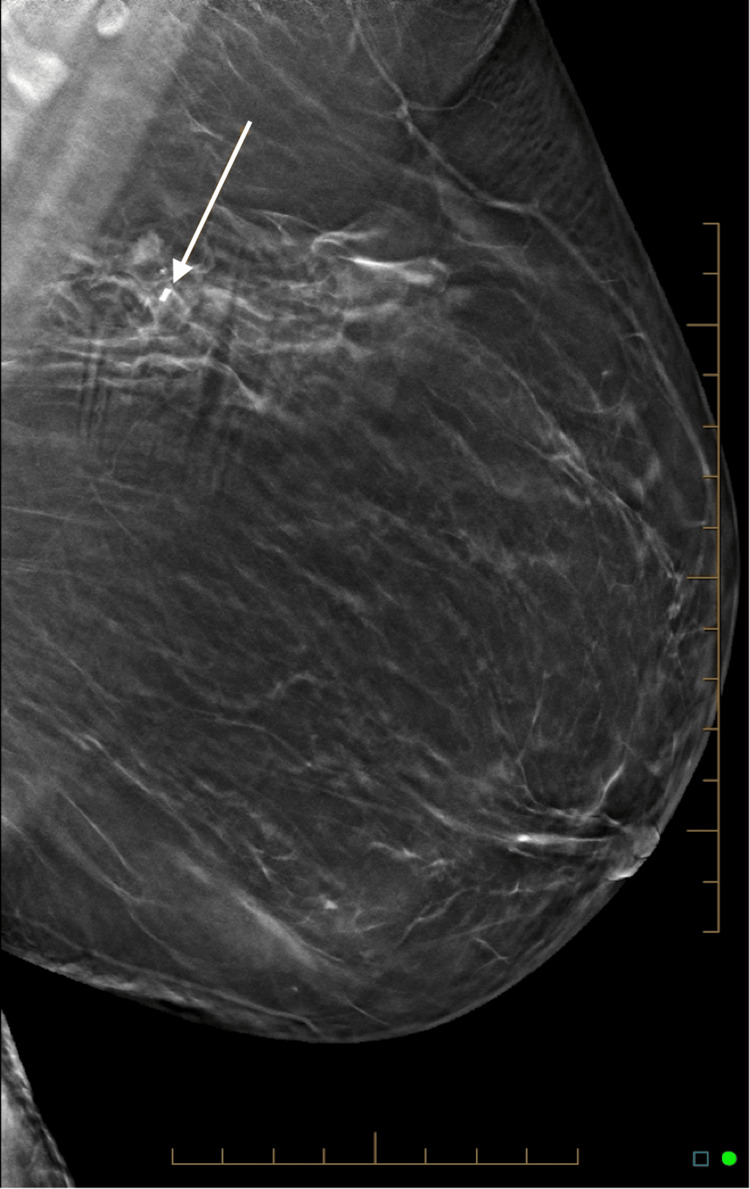
Post-interventional left mediolateral oblique mammogram confirming the marker at the target.

**Figure 6:**
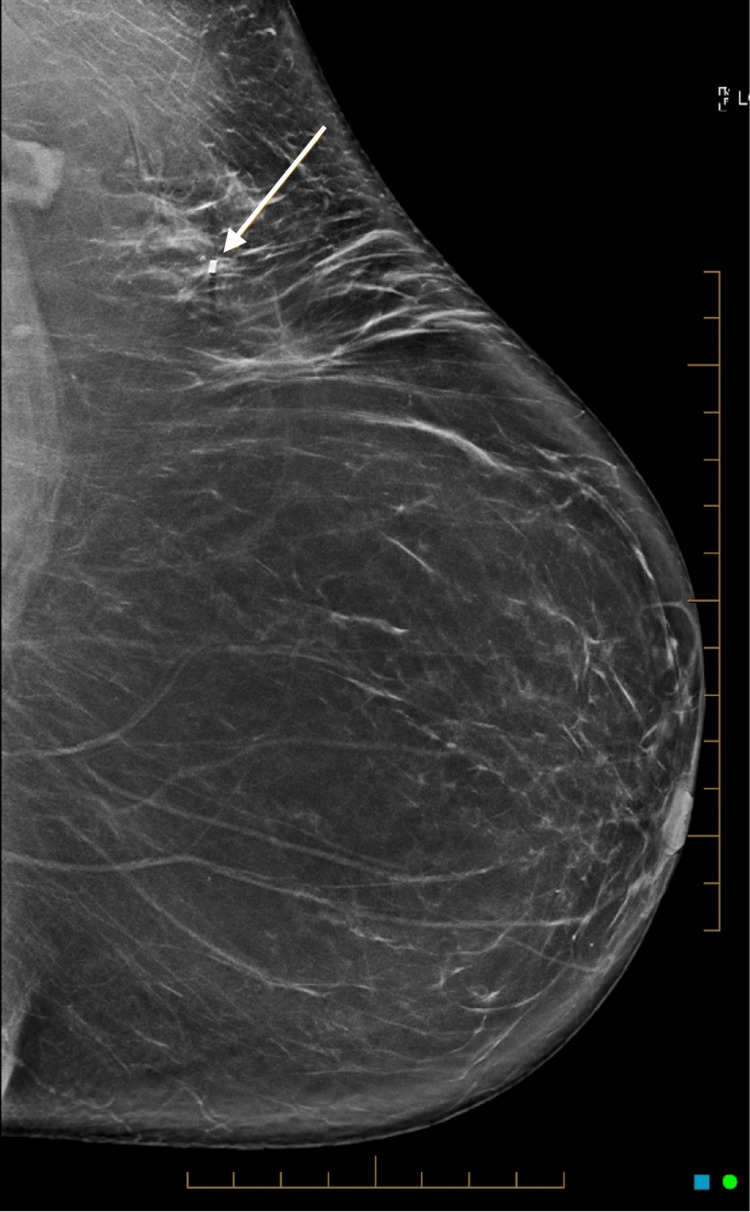
Left craniocaudal mammogram confirming the marker at the target.

A small titanium clip was placed at the end of the procedure. A post-biopsy two-view mammogram displayed the marker at the target (Figures 5-6).

**Figure 7:**
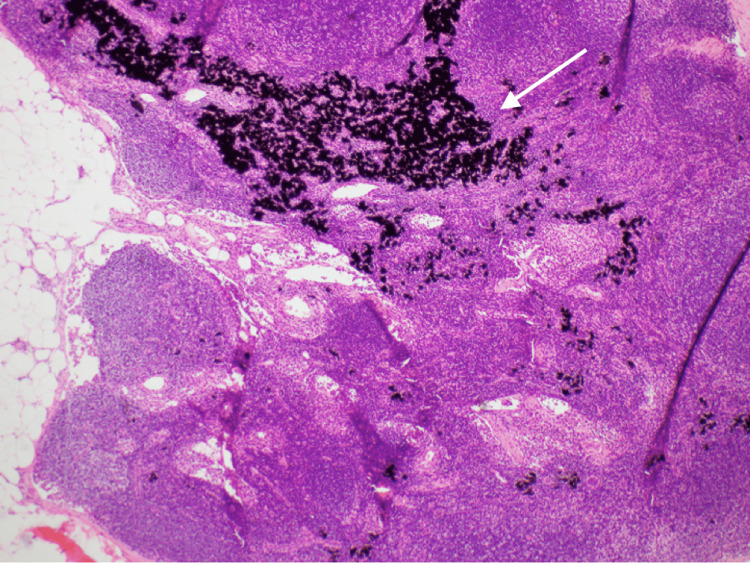
Low-power view of the black tattoo pigment within an otherwise normal lymph node.

**Figure 8:**
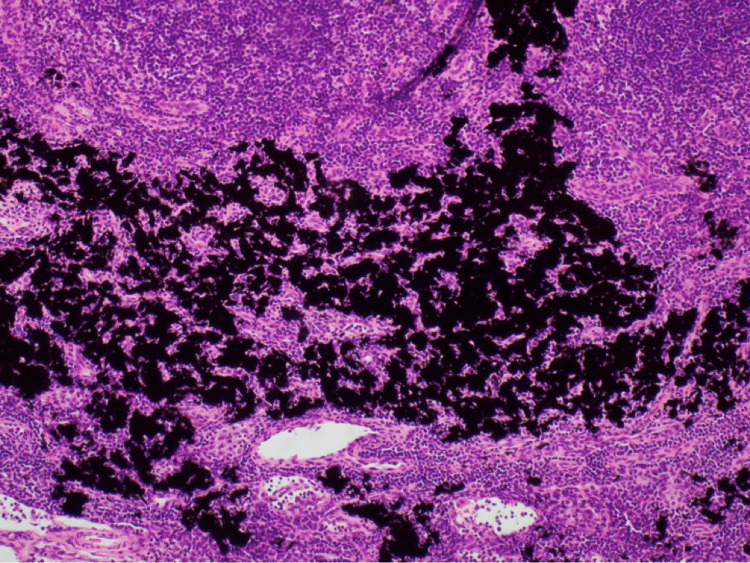
High-power view of black tattoo pigment within the lymph node.

Histopathology showed an intramammary lymph node with preserved nodal architecture. No evidence of lymphoid atypia was seen within the node. Prominent extracellular black pigment was identified, suggestive of tattoo pigment. No calcification was seen (Figures 7-8).

Thus, the initially presumed calcification within the breast lesion was identified as tattoo pigment in the intramammary lymph node. This correlated with the clinical observation that the patient had multiple tattoos on her left shoulder and upper arm.

## Discussion

Tattooing has become increasingly popular with an overall prevalence of 10-29% worldwide [2]. This popularity is mainly driven by the younger population group between 18 and 34 years of age [3,4]. In addition to this, the incidence of females having tattoos has quadrupled since the early 1990s. Whereas males have historically had significantly higher rates of tattoos, the prevalence of tattoos is now gender equal or even 5-10% greater for females in some countries such as Australia [3-5]. As these younger females start breast screening from 40 years of age, there will be continued growth in the prevalence of tattoos within the screening population [6].

Tattooing is a deposition of ink within the dermis via repeated puncture using 200-300 micron-sized needles [7]. Tattoo ink composition varies in quality but commonly consists of metallic salts with a carrier solvent such as water, alcohol, binders, or surfactants [8,9]. The choice of metals depends on the desired color, the artist is wanting to produce, for example, iron for black, mercury for red, cobalt for blue, and cadmium for yellow [10]. These materials make the tattoo pigment particles radiopaque and therefore they appear as calcifications on imaging including mammograms [11,12]. The skin injuries caused by repeated puncturing activate an inflammatory response, resulting in phagocytosis, vasodilation, and influx of monocytes [13]. Macrophages containing deposited ink either remain in the dermis, resulting in permanent tattoo visible on the skin or are excreted via lymphatic drainage [7,10].

The lymphatic drainage of skin varies by its location but also varies depending on the individual. Tattoo pigment from upper limbs will drain to ipsilateral sentinel nodes in the axilla 90-91% of the time, as well as into the internal mammary sentinel node in 1% of cases [14]. Lymphatic drainage from the trunk to intramammary lymph nodes has also been described in the literature [15]. There have been several studies that have reported tattoo pigment mimicking axillary lymph node calcifications [16,17]. To our knowledge, this is the first case report of pigment from an upper limb tattoo mimicking breast malignancy.

## Conclusions

Tattoo ink can mimic breast calcification as presented in this case. As cosmetic tattooing becomes more prevalent among the female population, tattoo pigment should be considered as a differential diagnosis for microcalcification seen on breast mammography. Further diagnostic workup will be required to differentiate between breast malignancy and tattoo pigment.
